# Quantum dot loaded immunomicelles for tumor imaging

**DOI:** 10.1186/1471-2342-10-22

**Published:** 2010-10-18

**Authors:** Aristarchos Papagiannaros, Jaydev Upponi, William Hartner, Dmitriy Mongayt, Tatyana Levchenko, Vladimir Torchilin

**Affiliations:** 1 Department of Pharmaceutical Sciences and Center for Pharmaceutical Biotechnology and Nanomedicine, 312 Mugar Life Sciences Building, Northeastern University, Boston MA 02115, USA

## Abstract

**Background:**

Optical imaging is a promising method for the detection of tumors in animals, with speed and minimal invasiveness. We have previously developed a lipid coated quantum dot system that doubles the fluorescence of PEG-grafted quantum dots at half the dose. Here, we describe a tumor-targeted near infrared imaging agent composed of cancer-specific monoclonal anti-nucleosome antibody 2C5, coupled to quantum dot (QD)-containing polymeric micelles, prepared from a polyethylene glycol/phosphatidylethanolamine (PEG-PE) conjugate. Its production is simple and involves no special equipment. Its imaging potential is great since the fluorescence intensity in the tumor is twofold that of non-targeted QD-loaded PEG-PE micelles at one hour after injection.

**Methods:**

Para-nitrophenol-containing (5%) PEG-PE quantum dot micelles were produced by the thin layer method. Following hydration, 2C5 antibody was attached to the PEG-PE micelles and the QD-micelles were purified using dialysis. 4T1 breast tumors were inoculated subcutaneously in the flank of the animals. A lung pseudometastatic B16F10 melanoma model was developed using tail vein injection. The contrast agents were injected via the tail vein and mice were depilated, anesthetized and imaged on a Kodak Image Station. Images were taken at one, two, and four hours and analyzed using a methodology that produces normalized signal-to-noise data. This allowed for the comparison between different subjects and time points. For the pseudometastatic model, lungs were removed and imaged *ex vivo *at one and twenty four hours.

**Results:**

The contrast agent signal intensity at the tumor was double that of the passively targeted QD-micelles with equally fast and sharply contrasted images. With the side views of the animals only tumor is visible, while in the dorsal view internal organs including liver and kidney are visible. *Ex vivo *results demonstrated that the agent detects melanoma nodes in a lung pseudometastatic model after a 24 hours wash-out period, while at one hour, only a uniform signal is detected.

**Conclusions:**

The targeted agent produces ultrabright tumor images and double the fluorescence intensity, as rapidly and at the same low dose as the passively targeted agents. It represents a development that may potentially serve to enhance early detection for metastases.

## Background

Near infrared (NIR) imaging is a particularly promising method of imaging since it is not invasive, requires relatively simple and easy-to-use equipment, and can take place in real time. The detection limit can be as low as in other imaging modalities, and it is much less hazardous compared to radionuclide or magnetic resonance imaging as it does not make use of radioisotopes that have special handling and storage requirements, both for their use and their disposal [1]. It is also very versatile and affordable. Instruments are not as expensive or complicated as those for nuclear or magnetic imaging [2]. In the NIR, the light penetrates much further through the body compared to imaging in the visible part of the spectrum [3]. This 'absorbance window' allows for the visualization of various phenomena deep inside the body. Using targeted contrast agents, the fluorescence signal can be highly localized. Examples include the detection of the epidermal growth factor receptor [4] and vascular endothelial growth factor receptors in mice [5], or cathepsin metal-proteases in early pancreatic cancer [3]. Detailed and precise images of anatomical and functional aspects of animals can be produced using actively targeted fluorophores [6,7], but a high signal-to-noise ratio is difficult to achieve, since NIR fluorescence is typically scattered throughout the tissues of mice [8]. Thus, the need for highly fluorescent targeted nanoparticles that will allow precise optical imaging with a high signal-to-noise ratio and minimal invasiveness and using simple instrumentation remains.

Quantum dots (QD) are semiconductor nanocrystals made of inorganic materials, such as CdSe. They exhibit nanosized dimensions and low polydispersity [9] Their excitation and emission spectra depend on their size, so that different emission spectra can be produced essentially from the same materials simply by changing their size. This allows simultaneous imaging of different aspects of a pathological site [10] Their advantages for imaging include bright fluorescence, excellent photo-stability, and variety in possible emission spectra [11-13]. They are ideal for optical imaging and considered reasonably safe, provided that a stable coating is applied during their manufacture [11,14]. Their applications include cell labeling [15], cell trafficking studies [16], sentinel lymph node imaging [17], lymphatic imaging [18], detection of apoptosis [19], tumor detection, and brain imaging [20]. NIR-emitting QD that exhibit a high molar excitation coefficient are particularly suited for the *in vivo *whole body imaging. They can allow the detection and tracking of single cells throughout the body [21]. Since their surface properties determine their biodistribution, various modifications of QD have been tested to achieve long circulation times and either passive or active targeting to areas of interest [22,23].

Lipid-core micelles are a versatile system for the administration of drugs, DNA or imaging agents [24] and they have proven to be a safe and highly biocompatible system [25-27]. They are composed mainly of amphiphilic block-copolymers composed of soluble blocks, such as polyethylene glycol (PEG), and insoluble lipid blocks, such as phosphatidylethanolamine (PE). They self-assemble when the concentration of the amphiphilic copolymers is above their critical micellar concentration, exhibit both high stability and excellent biocompatibility, and are very stable as long as their concentration in the plasma is higher than their critical micellar concentration. They have been used extensively for the delivery of potent but poorly soluble drugs for cancer therapy, like paclitaxel, or meso-tetraphenylporphin in photodynamic therapy [28,29]. They have also been used as contrast agent carriers for tumor imaging, such as QD carriers in whole body optical imaging, as carriers of super-paramagnetic nanoparticles for magnetic resonance imaging and with radionuclides, such as ^111^In, for gamma imaging studies [25].

Recently, we introduced a NIR contrast agent for whole body imaging composed of QD-loaded PEG-PE micelles (QD-Mic). The advantages of this formulation compared to commercially available formulations include the rapid accumulation of the agent at the tumor site (one hour compared to four hours for the commercial formulation) and sharply contrasted images. Using a novel quantification method for planar imaging, we were able to determine that QD-Mic doubled the signal at the tumor site with half the dose compared to passively targeted QD-Mic [30]. Thus, the discrimination of tumors and internal organs was possible. Here, we present the next step in the development of QD-Mic, by actively targeting QD-Mic to cancer cells. This was possible by the additional modification of the PEG-PE coat of QD-Mic, to which a broad variety of targeting ligands can be attached. In this study, we used QD-Mic modification with the monoclonal antibody (mAb) 2C5, which has a nucleosome-restricted specificity and recognizes a variety of cancer cells via cancer cell surface-bound nucleosomes released from the apoptotically dying neighboring cancer cells [31]. mAb 2C5-targeted micelles were shown to accumulate specifically in tumors and increase the anti-tumor effect of micelle-incorporated drugs [28]. Nucleosomes are abundant on the surface of all uncontrollably proliferative cancer cells. Recent reports from our lab demonstrate the use of mAb 2C5-labeled micelles or liposomes, both to established tumors and metastasis, including B16 melanoma tumors [32-34]. Such immunomicelles can attach to cancer cells even when the tumor is small, and may serve as a vehicle for the early detection and treatment of cancer micrometastasis [25]. Early detection (and treatment) of metastasis is of great importance for the cure of cancer, since the mortality is usually due to the development metastatic cancers and not to the primary tumor [35,36].

In this paper, we describe the development of 2C5-targeted QD-loaded PEG-PE-based immunomicelles that can detect small tumor sites.

## Methods

### Micelle characterization and stability

Micelle size was determined by the dynamic light scattering (Beckman Coulter N4 Plus, USA).

### Preparation of QD-Mic

Near infrared emitting CdSe QD (Qdots 800, Invitrogen, USA) were incorporated in micelles as previously described [25,37,38]. Briefly, a QD suspension in decane was mixed with a four-fold volume of a 1:3 isopropanol/methanol mixture and centrifuged at 3500 rpm for 5 min. The supernatant was discarded and the QD pellet was re-suspended in chloroform. For micelle production, 2.7 μmoles of PEG2000-PE [N-carbonyl-methoxy-poly-(ethyleneglycol-2000)-1,2-distearoyl-3-phospho-ethanolamine sodium salt] containing a 5% molar ratio of the reactive para-nitrophenylcarbonyl(pNP)-PEG2000-PE was mixed with 20 pmoles of QD in chloroform, and solvents were evaporated under vacuum. The system was freeze-dried overnight and, when necessary, hydrated in phosphate buffered saline (PBS), pH 7.4, or citrate buffer, pH 5.0. Micelle size was determined.

### Conjugation of 2C5 antibodies to QD-Mic

The PEG-PE/QD mixture was hydrated in 200 μl of the citrate buffer, pH 5.0, with vortexing. After an equilibration period of 1 hour, the micelles were incubated in a 3-fold excess borate buffer, pH 9.3, and mixed with a 2-fold excess of 2C5 antibody (339 μl of a 2.84 mg/ml solution). They were then dialyzed (cut-off value MW 250,000) overnight against PBS, pH 7.4 at 4°C.

### Tumor inoculation in mice

Female Balb/c mice, 6-8 weeks old, (Charles River Laboratories, Wilmington, MA) were inoculated with tumors following a protocol approved by the Northeastern University Institutional Animal Care and Use Committee in accordance with the 'Principles of Laboratory Animal Care' (NIH publication No. 85-23, revised in 1996). The 4T1 murine breast cancer cells were grown in Dulbecco Modified Eagle's Medium (DMEM) supplemented with 10% fetal calf serum. Cells (1.5 × 10^5 ^per mouse) were suspended in 150 μl of PBS and injected subcutaneously in the right flank. Tumors developed within two weeks after the injection. The animals had free access to food and water.

### Pseudometastatic melanoma model

B16F10 cells were grown in DMEM supplemented with antibiotics and 10% fetal calf serum. Cells (8 × 10^5 ^per mouse) were injected in the tail vein. In approximately two weeks, cancer cell nodes developed in the lungs of the animals. Control animals were sacrificed by carbon dioxide euthanasia, lungs removed, washed in saline, and the melanoma nodes were counted. *Ex vivo *white field photographs of the lungs were taken using the Kodak Image Station In Vivo FX (Carestestream Health, USA). Cancer cell nodes were easily recognized as the black spots on the lung surface.

### Near Infrared mouse imaging

Contrast agents, 40 pmoles/mouse[11] were injected via the tail vein. For the pseudometastasic model, 1/10 of the above dose was injected. After the administration of the contrast agents, mice were anesthetized (ketamine/xylazine, i.p.) and depilated using a hair removal cream (Nair, USA) prior to tumor observation. Images were taken with a Kodak Image Station (filters: excitation 710 nm, emission 790 nm) at different angles. At the end of the experiment, mice were sacrificed with CO_2_, skin removed to avoid its scattering of the fluorescence, and images were retaken to precisely localize the internal organs. All images were analyzed using the Kodak Image Analysis software or the ImageJ (NIH, USA). The regions of interest (ROI) were determined using threshold analysis by comparing the whole body images with the images after the skin was removed. The mean pixel intensity, the background signal and the auto-fluorescence of the animal was extracted at each time point. The mean pixel intensity at the ROI was expressed as an absolute number by comparison with the scattered light (background noise from the internal organs near the ROI), and all images were normalized using a ROI over the hip of the mouse to compensate for differences between the NIR images of the different animals and time points that result from differences in the overall image luminosity.

### Detection of cancer cell nodes in the pseudometastic model

Contrast agents were injected as previously described. At one hour and twenty four hours after the injection of the contrast agent, animals were sacrificed and lungs removed. Images of the removed organs were taken as before, both in white field and in near infrared.

## Results

### Micelle size

The size of the 2C5 QD-Mic was 21.0 ± 6.7 nm and QD-M 17.6 ± 1.2 nm. Micelles were stable at 4°C for at least fifteen days.

### Signal-to-noise ratio

**Figure 1:**
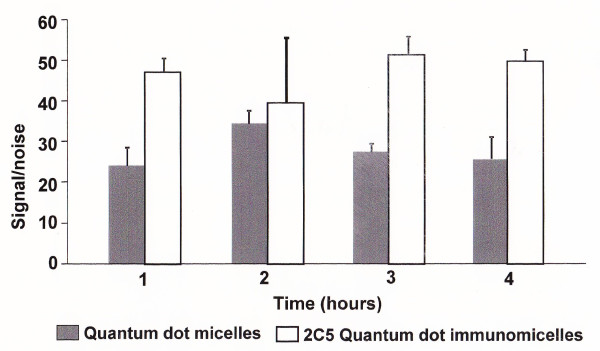
**Normalized signal-to-noise ratio for the fluorescence of the tumor**. Data show the ratio of the tumor fluorescence vs. the background in the area around the tumor for 2C5 modified QD-Mic and for QD-Mic without antibody. Error bars represent standard deviation, N = 5.

Figure 1 shows the signal-to-noise for the 2C5-QD-Mic at the area over the tumor over 4 hours. The signal-to-noise is high within the first hour and remains practically unchanged for the duration of the imaging. One hour after the injection, the signal-to-noise was 47.1 ± 3.1, while at the end of imaging it was 49.8 ± 2.7. This signal was double that of the non-targeted QD-Mic and almost four times higher that of the commercially available formulation of PEG grafted quantum dots (Qtracker, Invitrogen, USA) administered at double the dose published earlier [30].

### Biodistribution of the 2C5-QD-Mic using image analysis

**Figure 2:**
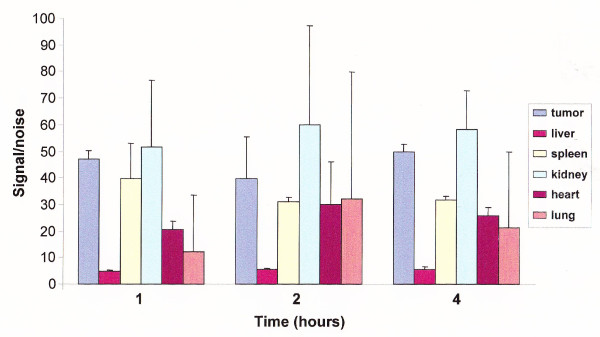
**Quantification of the biodistribution of 2C5 QD-M in animals**. Units represent net light intensity in the near infrared region weighted vs. the autofluorescence of the mouse. Error bars represent standard deviation, N = 5.

Figure 2 shows the distribution of the NIR signal to the various tissues. The methodolody, as published earlier, involves the generation of weighted signal-to-noise data for the duration of the imaging experiment. The signal-to-noise is comparable among the different time points, as it represents normalized values with reference to the intrinsic fluorescence of the animal. This analysis allowed us to express and quantify the imaging effect of the contrast agent *in vivo *[39]. The highest signal was detected in the tumor area (47.2 ± 3.1 at one hour after the injection) and the kidney (51.4 ± 25.2). The rest of the organs exhibited a lower signal, the lowest of which was from the liver with the signal intensity of 4.97 ± 0.26. This pattern remained unchanged throughout the imaging period, so that at four hours after the administration of the contrast agent, the signal in the tumor area was 49.8 ± 2.7. The liver again exhibited the lowest signal of 5.64 ± 0.82. With the exception of the kidney and spleen, the actively targeted 2C5 QD-Mic persisted in the tumor area with lower affinity for other organs.

### Composite images of mice injected with 2C5-QD-Mic

**Figure 3:**
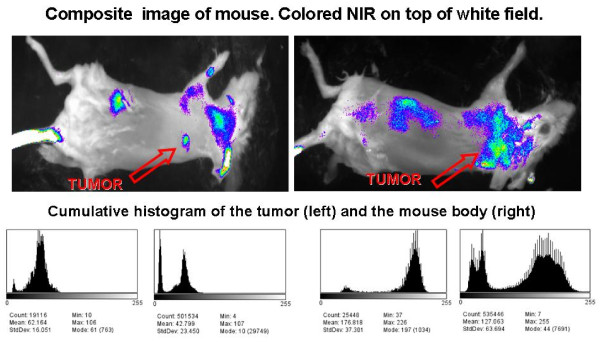
**Composite images (white field image superimposed with the fluorescence intensity), and cumulative histograms for the tumor region and the whole body of two mice (A and B) injected with 2C5 QD-Mic**. Fluorescence is concentrated mainly in the tumor area, and the cumulative histograms of the frequency of pixels vs. their value for the tumor area and the body of the animal verify that the region of interest (tumor area) has a narrow distribution of the highest value pixels from the animal body.

Figure 3 shows composite NIR images of two mice injected with 2C5-QD-Mic one hour after the injection, superimposed over a white field image. The signal is visible only from the tumor area, indicated by the arrow. Some signal was detected from hairs that were not completely removed. The histograms of the pixel values (Figure 3) verified this conclusion. Pixel values for the tumor area had the highest values compared to the rest of the animal body. For instance, the mean value in the non-tumor ROI is 42.8 ± 23.5, and 62.2 ± 16.1 in the tumor area. It is of particular interest that the pixel distribution is much narrower in the tumor ROI. The high slope of the pixel value distribution allowed the tumor to be identified clearly.

### Composite ex vivo images of lungs from mice injected with 2C5-QD-Mic in a melanoma pseudo-metastatic model

**Figure 4:**
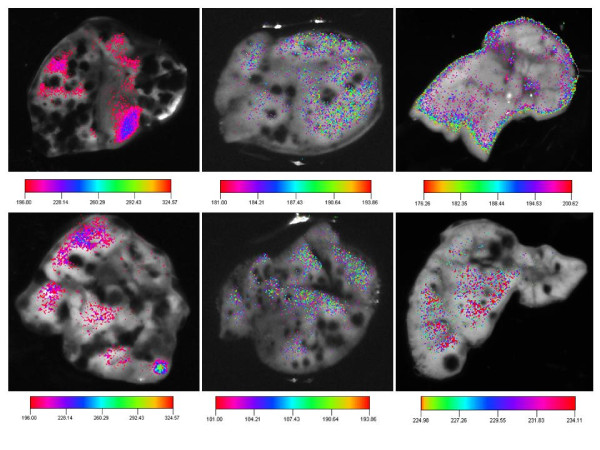
**Composite *ex vivo *images (white field image superimposed with the fluorescence intensity) of lungs from mice bearing metastatic B16F-10 lung melanoma tumor**. Left: two mice not injected with 2C5 QD-Mic. We see the clusters of melanoma cells (black) in the lungs (white) and a low level of near infrared fluorescence. Center: two mice injected with 2C5 QD-Mic at one hour after the injection. There is a higher level of near infrared fluorescence from the lungs. Right: two mice injected with 2C5 QD-Mic at twenty-four hours after the injection. The fluorescence originates mainly from sites around the melanoma clusters allows detection of the metastatic sites.

In Figure 4, B16F10 melanoma cells appeared black, while normal cells are white, since Balb/c mice are albino. The whole body images from control mice injected with B16F10 melanoma cells, but not the 2C5-QD-Mic represents the intrinsic fluorescence of the organ and showed a low level of uniform NIR fluorescence. The cancer cell clusters were clearly identifiable by their black color. The background fluorescence was uniformly distributed over the lungs. A large number of cancer cell nodes could be seen in the white field photograph. One hour after the injection of the 2C5-QD-Mic, the NIR fluorescence was still diffuse over the entire lungs with a pattern similar to the background fluorescence but with a slightly higher intensity. At 24 hours after injection, NIR fluorescence was much less uniform. Signal was concentrated mainly at areas near the small clusters of cancer cells and in the periphery of the lungs, pinpointing the smaller cancer cell clusters.

## Discussion

The need for efficient contrast agents surfaced with the development of practical and easy-to-use instruments for NIR whole body imaging. Lipid-based coatings for QD were proposed by us and by other labs involves encapsulation of one quantum dot within a lipid envelope [37,38,40,41]. Our approach described aimed to alter the biodistribution profile of QD to allow for effective and relatively rapid imaging of tumor sites. With this in mind, we have developed a contrast agent that actively targets tumor sites and results in a high signal-to-noise ratio. This formulation represents a significant improvement over both the commercially available pegylated QD such as the Qtracker^®^, and passively targeted QD-containing PEG-PE micelles earlier described [30], since it allows for rapid imaging using a low dose of QD (half that of the Qtracker^®^) and with a two-fold enhancement of image intensity compared to non-targeted QD-Mic at the same QD dose. The encapsulation of QD in a PEG-PE envelope allows for the easy attachment of antibodies and other ligands without using complex surface chemistry [40,41]. The attachment of antibodies was performed via the micelle-incorporated reactive pNP-PEG-PE component, which is simple and straight-forward compared to the use of an avidin-biotin linker that exposes the complex to opsonization by blood proteins and may decrease its circulation time [42]. This method also offers advantages such as simplicity and high stability over systems composed of multiple cores in a complex lipid system [43].

Previously, we showed that the encapsulation of QD into the PEG-PE envelope increases the signal of QD in the tumor area compared to unmodified "native" QD, decreases the necessary imaging time from four hours to one hour, and allows for a decrease of the equivalent dose of the imaging agent by one half. The attachment of the anti-tumor 2C5 antibody further doubles the signal by bringing more contrast agent into the tumor, similar to earlier demonstrated enhanced imaging by 2C5-targeted contrast agents with other imaging modalities [44]. This targeting allowed us to image the tumor site with a superior signal especially to that of the liver. This increase in the signal of 2C5 QD-M was also reported in a variety of earlier papers from our lab using the anti-nucleosome 2C5 anticancer antibody which is generic to all uncontrollably proliferating cell lines [29]. The effect of targeting has been demonstrated in various systems, including doxorubicin liposomes modified with the post-insertion method [33] and micelles [45] and using different imaging modalities such as MRI [44] or gamma imaging [46].

Antibodies are well known agents that increase the accumulation at sites overexpressing tumor antigens [47,48] The attachment of antibodies permits the targeting and visualization of surface antigens in low concentrations [49] and especially micrometastasis [50]. High affinity antibodies saturate the surface of the tumor and it becomes difficult to access the tumor mass, thus the enhanced permeation effect can become critical [51] and in fact, this effect, was recently modeled stochastically [52-55]. Nanoparticles accumulate mainly through passive targeting while the tumor localization of the nanoparticles is influenced by the antibody [56]. The antibodies therefore are essential for the specificity of the cell targeting while the passive accumulation is the main force for the biodistribution of the nanoparticles to the solid tumor [57]. This is a proof of the versatility of our imaging methodology, that produces data as normalized signal to noise, which quantify the imaging effect (contrast) rather than simply the amount of the nanocarrier present at the tumor site. This was also done by other groups, expressing the targeting effect as a two-fold increase over the passive accumulation, using radioactivity and calculating the ratio of the tumor vs. the hip muscle [58].

The use of lipid-based nanoparticles for delivery of QD has recently gained a lot of attention. A hybrid QD/cationic liposome system was recently assembled [59]. This system significantly enhanced the delivery of QD in tridimensional cell culture systems, due mainly to the effect of the positive charge of the nanoparticles. Another system used conventional immunoliposomes [60] with QD attached via PEG spacers at the surface of the nanoparticles. This latter system offers bright images due to the presence of a large number of fluorophores per nanoparticle. The simplicity of the 2C5-QD-Mic may provide an advantage. 2C5-QD-Mic represent an improvement over QD directly conjugated to an antibody, where the QD antibody complex lacks the steric protection provided by PEG coating [61].

The greater efficiency provided by the higher signal within a shorter time should be of great benefit for cancer imaging. Recent studies have shown that QD toxicity may be low enough for systemic administration [62]. The small number of studies on the use of QD for the detection of metastases [62-64] may be attributed to the low sensitivity of the optical imaging and the intense scattering of the light that makes pinpointing of the source and estimation of the size of small tumors difficult. The clear benefit of our formulation is its high affinity towards cancer cells introduced by the 2C5 antibody [28,65]. Although the period of 24 hours for the detection of metastases is longer than that of the imaging of a larger tumor (one hour), this is due to the need to allow the contrast agent long enough time to circulate and attach in sufficient quantity to the cancer cells in loci containing small numbers of such cells. This observation also implies that both passive targeting and antibody-mediated attachment to the tumor are involved in the accumulation of the targeted contrast agent at the tumor site. The lung tissues are first saturated with the contrast agent and the attachment of 2C5-QD-Mic to cancer loci follows together with the elimination of the unbound contrast agents from the lungs as a whole. The insufficient staining of bigger cancer masses may be explained by an absence of 2C5-QD-Mic penetration of the tumor cells.

The use of NIR optical imaging for the detection of metastases has gained more attention recently and is a useful modality for the intra-operative detection of lymph node metastases [66]. By administering quantum dots before the operation the lymph nodes can be visualized during the operation for the removal of a breast cancer and reduce the probability for the development of metastases. Optical methodology has obvious advantages, due to the simplicity of the instruments involved, for use in the operating room [67-70]. This imaging effect is in accordance with observations that the main thrust for tumor accumulation is the EPR effect while the antibody plays a helping role, which is limited to cell penetration, especially by large lipid nanoparticles such as liposomes. In fact, in many cases the antibodies stain only the periphery of the cell cluster and are unable to penetrate deeply inside the tumor mass. The net effect is a resultant drainage of the nanoparticles from the tumor [52,56].

However, as a modality for the detection of metastasis, near infrared imaging has limits, because of the low sensitivity of the method and the inability to detect cancer clusters in whole body imaging. Recent developments involving the quantification of the signal and the detection of fluorescence deep inside the body may make this type of imaging possible and useful [71]. Although the time necessary for imaging may be longer with QD-Mic than with some radioimaging contrasts, the benefits of using non-radiation emitting agents remain important. As evident from our data, there is a sufficient difference from the background signal of the healthy part of the lungs to permit localization of cancer cells within healthy organs. Our system may be useful to allow the detection of a signal from remote sources throughout the body and permit visualization of small clusters of cancer cells before they develop into apparent tumors [72], especially if coupled with an advanced imaging modality that captures *in vivo *images in real time [73,74].

## Conclusion

In summary, 2C5-QD-Mic allow imaging as equally rapid as the non-targeted micelles and produce twice the signal at the same dose. Their production is easy and requires no special equipment. With the development of advanced imaging instruments that allow signal detection deep within the body, their potential usefulness can be expected to be significantly enhanced.

## Competing interests

The authors declare that they have no competing interests.

## Authors' contributions

AP, Conception and design of the contrast agents and the image analysis methodology, execution of experiments and draft of the paper, JU contrast agent preparation and animal imaging, DM. 2C5 antibody conjugation, WH animal experiments, TL and VT conception and design of the study. All authors read and approved the manuscript.

## Pre-publication history

The pre-publication history for this paper can be accessed here:

http://www.biomedcentral.com/1471-2342/10/22/prepub
